# Neural systems underlying autobiographical memory dysregulations in depression: A neuroimaging meta-analysis

**DOI:** 10.1017/S0033291725102833

**Published:** 2025-12-29

**Authors:** Cheuk Chi Charlotte Cheng, Michelle Hei Lam Tsang, Mengfan Han, Jie Zhang, Michael Maes, Benjamin Klugah-Brown, Mercy Chepngetich Bore, Benjamin Becker

**Affiliations:** 1 Department of Psychology, MIND & AI Lab, The University of Hong Kong, Hong Kong SAR, China; 2 School of Life Science and Technology, University of Electronic Science and Technology of China, Chengdu, China; 3 Institute of Science and Technology for Brain-Inspired Intelligence, Fudan University, Shanghai, China; 4 Sichuan Provincial People’s Hospital: Sichuan Academy of Medical Sciences, Chengdu, China

**Keywords:** amygdala, anterior cingulate, autobiographic, default mode network, depression, fMRI, hippocampus, meta-analysis, memory, salience network

## Abstract

Autobiographical memory (AM) dysfunction has been proposed as a neurocognitive mechanism underlying the development and maintenance of depression. However, case–control neuroimaging studies investigating the neural correlates of AM in depression have yielded inconsistent findings. The present study utilized neuroimaging meta-analyses to identify robust neural markers of AM dysfunction in depression and characterize the associated behavioral and network-level mechanisms. A preregistered neuroimaging meta-analysis (https://osf.io/35xtf) was conducted, incorporating data from 341 patients with unipolar depression, 82 individuals at risk of depression, and 261 healthy controls across case–control functional magnetic resonance imaging studies examining AM processing. Meta-analytic network-level and behavioral decoding analyses were performed to aid interpretation of the findings. Compared with controls, the depression group displayed increased activation in the right paracingulate cortex (dorsal anterior cingulate [dACC]) and precuneus, and decreased activation in the anterior insula during AM recall. Exploratory valence-specific analyses revealed that negative AM recall was associated with increased activity the dACC and precuneus. Meta-analytic decoding linked the dACC to the salience network and to domains related to negative affect and executive control, while the precuneus was associated with the default mode network and with processes related to social cognition and AM. Findings do not support prevailing models emphasizing altered amygdala and hippocampal function in AM deficits in depression. Instead, they highlight the involvement of core regions within the salience and default mode networks as key neural substrates of AM dysfunction. These regions may contribute to affective, social-cognitive, and mnemonic disturbances that shape the valence-specific nature of AM deficits in depression.

## Introduction

Autobiographical memory (AM) is a memory system that consists of episodes recollected from an individual’s life, that is, personal semantic and episodic information (Tulving 1972, 1983), with the former referring to facts about the self, such as information on where the individual was born, and the latter referring to unique events, such as the first time riding a bicycle (Brewer & Rubin, 1996). Dysfunctions such as impaired or biased AM recall have been observed across mood and anxiety disorders (Lenaert et al., 2015; Moscovitch et al., 2018; Weiss-Cowie et al., 2023), with accumulating evidence for an important role in the development and maintenance of unipolar depression (Weiss-Cowie et al., 2023). AM dysfunction in depressed individuals has been characterized by generally impaired recollection, as well as valence-specific effects, that is, impaired memory for positive events in the context of a better memory for negative events (Burt et al., 1995; Matt et al., 1992). Within the context of cognitive models of depression such as Beck’s negative cognitive triad model (2008; Beck et al., 2024) proposing that negative beliefs about the self, the world, and the future shapes depression, a negative AM bias can subserve a vicious cycle of dysfunctional and self-enforcing negative schemas that contribute to the development and maintenance of depression.

In experimental settings the behavioral and neural basis of AM and its alterations in mental disorders have been examined by combining the retrieval of general and emotional autobiographical memories, for example, by cued recall, personalized scripts or prospection (St. Jacques & Cabeza, 2012) with functional magnetic resonance imaging (fMRI, for details on the specific experimental paradigms, see also Brewer & Rubin, 1996; Gilboa, 2004; Young et al., 2012; Young, Bellgowan, Bodurka, & Drevets, 2013). Despite a certain level of inconsistency between the studies, recent meta-analyses confirm impairments in retrieving specific autobiographical memories in individuals with depression and increased risk for depression (Hallford et al., 2021, 2022; Liu et al., 2013), with some original studies suggesting valence-specific effects, for example, better memory for negative events (Kuyken & Dalgleish, 2011).

The neurobiological basis of AM has been examined in numerous fMRI studies suggesting that AM relies on circuits encompassing prefrontal regions as well as the hippocampal formation, retro splenial cortex and posterior cingulate cortex (PCC; Shepardson, Dahlgren, & Hamann, 2023; Svoboda et al., 2006), with some evidence for differential involvement of the basal ganglia and amygdala/insula depending on the emotional content of the memories (Testa et al., 2024). The widespread engagement may reflect the complex interaction between mental processes during AM encompassing memory recall, self-referential processes, executive function, imagery and semantic contextualization. Within this network the hippocampal formation plays a pivotal role in the memory formation and recall (Fink et al., 1996; Gardini et al., 2006; Greenberg et al., 2005; Ryan et al., 2001) as well as in detailed and immersive recollection of memory details for AM, and is further supported by the medial prefrontal cortex (PFC) involved in the processing of self-referential stimuli (St. Jacques & Cabeza, 2012). Furthermore, the lateral PFC (specifically the left ventrolateral PFC) has been identified as a key region for memory search and retrieval in AM recall (Shepardson et al., 2023). Regions such as the amygdala or the basal ganglia have been identified to mediate the emotional content of AM, such that the amygdala has been involved in recalling negative events (Doré et al., 2018; McCrory et al., 2017) or the subjective sense of remembering visual (Greenberg & Rubin, 2003) and emotional (Rubin & Berntsen, 2003) re-experiencing of the event (Piolino et al., 2009). Affectively colored AMs are evoked by stimulation of the amygdala (Vignal et al., 2007), and the activity of amygdala single neurons were associated with familiarity and recollection (Rutishauser et al., 2008), while the globus pallidus may mediate the emotional experience of positive AM (Testa et al., 2024).

More recent approaches examining the network-level underpinnings of AM further indicate that the default mode network (DMN) – a large-scale brain network associated with self-referential processes, mind wandering, and memory (Faustino, 2022; Philippi et al., 2015) plays a significant role in AM. A recent fMRI meta-analysis on AM by Shepardson et al. (2023) found that there is a large degree of overlap between the DMN and brain regions involved during AM retrieval, particularly in ‘core DMN’ regions, including frontal and posterior cortical midline structures and the bilateral angular gyrus (Andrews-Hanna, Smallwood, & Spreng, 2014; Shepardson et al., 2023).

While fMRI meta-analyses have allowed to robustly determine the neural systems underlying AM in healthy individuals (Shepardson et al., 2023), results on the neural systems mediating dysfunctional AM in depression are based on single studies that are characterized by a level of inconsistency inherent to case–control fMRI studies (Etkin, 2019; Klugah-Brown et al., 2021; Köhler et al., 2015). The development of neuroimaging meta-analytic approaches facilitates quantitative integration of findings from case–control MRI studies, offering critical advancements toward determining more robust structural and functional alterations in depression within a behavioral domain or in comparison to other mental disorders (Bore, Liu, Huang, et al., 2024; Liu et al., 2022; Zhou et al., 2020).

Against this background, the present preregistered neuroimaging meta-analysis aims to compensate for single-study deficits in aspects such as sample size and specific AM recall tasks that differ across studies. Neuroimaging meta-analyses have been developed to pool data extracted from original studies, which allows for examination of brain activity across studies with a higher statistical power. As such a neuroimaging meta-analysis on AM recall in depressive and at-risk individuals can increase the robustness and generalizability of the results and allow to quantitatively determine common brain regions associated with AM recall in depressive individuals. Similar approaches have been recently applied to other domains and have indicated, for example, robust striatal alterations during reward processing in depression across domains and at-risk populations (Bore, Liu, Gan, et al., 2024; Bore, Liu, Huang, et al., 2024; Zhou et al., 2024). These findings may also help to inform interventions that aim at targeting neural alterations in depression, for example, by closed-loop real-time neuroimaging interventions (Li et al., 2019; Misaki et al., 2024; Young, Siegle, et al., 2017).

Based on previous studies, we hypothesized that (1) neural alterations during AM in depression will be observed in the hippocampus and amygdala, in particular decreased activity in the hippocampus, and increased amygdala response in depressive individuals when retrieving negative autobiographical memories (eg, Doré et al., 2018; Young, Bodurka, and Drevets, 2016; Young, Siegle, Bodurka, & Drevets, 2016) and that (2) the identified regions will connect on the network level with the DMN.

To test our hypotheses we capitalized on previous case–control neuroimaging studies on AM in depression and performed a coordinate-based meta-analysis on Seed-based d mapping with Permutation of Subject Images (SDM-PSI), a novel and robust meta-analytic technique that produces unbiased estimation of effect sizes and generation of neurofunctional maps (Albajes-Eizagirre et al., 2019), with subsequent meta-analytic co-activation and connectivity analyses determining the network-level communication of the identified regions.

## Methods

### Search strategy and selection criteria

The current preregistered meta-analysis adhered to the guidelines of conducting a coordinate-based meta-analysis. A pre-registration was submitted on the Open Science Framework (https://osf.io/35xtf) platform to increase accountability and transparency prior to the commencement of the meta-analysis. A comprehensive literature search was conducted independently according to the Preferred Reporting Items for Systematic Reviews and Meta-Analyses (PRISMA) guidelines by C.C.C.C. First, a broad search of original fMRI studies on fMRI case–control studies AM in depression was conducted on PubMed (https://pubmed.ncbi.nlm.nih.gov/) and Web of Science (https://www.webofscience.com/wos/). Suitable studies from the reference lists of review articles were additionally included. The literature was screened referring to the returned titles and abstracts. Studies that were written in English, reporting whole-brain results and were published between 1998 and 2023 were included. Only peer-reviewed, original case–control studies comparing patients and healthy controls were included. The following search items were applied: ‘functional magnetic resonance imaging OR fMRI’ AND ‘autobiographical memory’ AND ‘depression OR major depressive disorder OR at-risk of depression’ have to co-occur with any of the following keyword: ‘memory recall.’ Data selection process was double checked by H.L.M.T. and M.C.B., with any discrepancies settled by B.B. This search yielded 150 unique studies as shown in the PRISMA flow diagram freely available (Page et al., 2021; https://www.prisma-statement.org/prisma-2020-flow-diagram), see Supplementary Table S2. The systematic literature review identified 13 suitable studies.

### Coordinate-based meta-analytic approach

A coordinate based meta-analysis of AM in individuals with or at the risk of depression was conducted using SDM-PSI (https://www.sdmproject.com) which performs statistical inferences on results from multiple studies (Albajes-Eizagirre et al., 2019) and has been successfully employed to determine robust neurofunctional alterations within or across mental disorders (Bore, Lui, Huang, et al., 2024; He et al., 2025). Peak coordinates and effect sizes of case–control differences during AM were extracted from the original studies reporting results on the whole-brain level. The meta-analysis additionally included results from studies in populations at the risk of depression. These studies commonly defined at risk as high familial risk for depression, that is, having a first-degree relative with major depression (Macdonald et al., 2016; Young et al., 2013; Young, Bellgowan, Bodurka, & Drevets, 2015; Young, Siegle, et al., 2016). Given that previous studies reported that AM impairments can be observed in patients with major depression disorder (MDD), at-risk individuals and in individuals with remitted depression (eg, Köhler et al., 2015; Kuyken & Dalgleish, 2011; Weiss-Cowie et al., 2023) and preliminary results indicate overlapping neural alterations (eg, Young et al., 2013). Hence, this study pooled such studies. The low number of studies including at-risk individuals (4), however, did not allow to run comparative meta-analyses. This practice was in line with previous meta-analyses, which article searches included both at risk and depressed patients (Bore, Liu, Gan, et al., 2024; Bore, Lui, Huang, et al., 2024).

Our major aim was to identify brain functional alterations during AM recall and its directionality in depression by implementing the Seed based d mapping extractions and mean analyses described in Supplementary Methods. *Z*-values representing between-group differences of depressed or at-risk individuals and healthy controls were transformed to *t*-values using the SDM statistical converter (https://www.sdmproject.com/utilities/?show=Coordinates). MNI coordinates (*x*, *y*, *z*) of activation clusters with statistically significant differences across tested contrasts will be reported.

Supplementary analyses were conducted on Neurosynth to explore the functional connectivity and meta-analytic co-activation patterns of the identified regions. Functional connectivity reflects brain regions that are coactivated across the resting-state fMRI time series with the seed voxel (Yeo et al., 2011), while meta-analytic coactivation employs all of the fMRI studies available in the Neurosynth database and serves as the meta-analytic analogue of the functional connectivity map (Rottschy et al., 2013; Schnellbächer et al., 2020) (details in Supplementary Methods).

### Analyses of the effects of valence AM recall

Given the frequently reported valence-specific effects in depression (Köhler et al., 2015; Liu et al., 2021), we conducted further exploratory analyses (Whalley et al., 2012; Wu et al., 2020). To identify possible effects of valence AM on brain region activation in AM recall, three sub analyses were performed based on evidence from the literature on valence effects on brain activity – one with all types of valenced AM recall, one with positive AM recall, and one with negative AM recall (McCrory et al., 2017; Young et al., 2012, 2013, 2014) (see Supplementary Methods).

### Analyses of the effects of depression risk in AM recall

Eighty-two people at risk of depression were included in our main analysis to increase the statistical power of our meta-analysis. However, to ensure that our results were not driven by at-risk groups, we also ran a separate analysis with depressed patients. This led to a subanalysis that included 12 studies, with one study being fully excluded (MacDonald et al., 2016) and some datapoints from 2 studies were further excluded (Young et al., 2013, 2015).

### Additional analyses

Using SDM values and other confounding variables such as age and sex, we performed meta-regression analyses.

### Tests for heterogeneity and publication bias

Heterogeneity and publication bias tests were performed with standard procedures. Publication bias was statistically evaluated by Egger’s test and funnel plots, where p values <0.05 were interpreted as significant.

## Results

### Demographic and clinical data summary of all included studies

**Figure 1.**
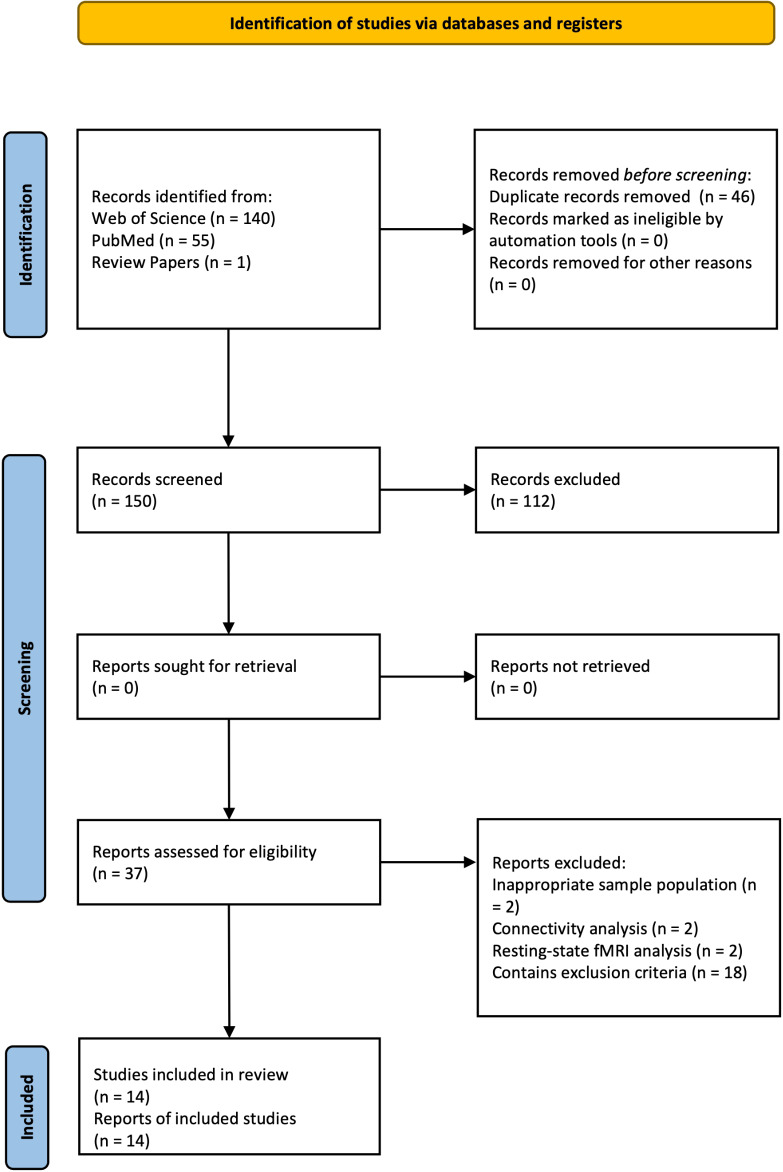
PRISMA flow diagram showing the identification of included studies.

A total of 13 studies comprising of 602 participants (*n* = 261 healthy controls, *n* = 341 patients) were included in the meta-analysis. There were 12 unique participant pools identified, as Young et al. (2013); Young, Bodurka et al. (2016) used the same participants in two separate studies. With exception of some studies which did not provide sufficient information (Gillard et al., 2023; MacDonald et al., 2016; Whalley et al., 2012; Wu et al., 2020), participants had a mean age of 34.41 (*SD* = 10.761). Healthy controls (*n* = 156) had a mean age of 34.17 (*SD* = 11.169), and depressive participants (*n* = 384) (including high risk, depressive and remitted depressive patients) had a mean age of 34.52 (*SD* = 10.604). There were no significant group differences between healthy controls and patients (*t* = .342, *p* = .732). The prisma flow diagram as well as a complete list of included studies and their design characteristics are shown in Figure 1 and Table 1.

**Table 1.** Demographic and clinical characteristics of included studies

Study	N				Control task	Active task
	HC	MDD	rMDD	HR		
Foland-Ross et al. (2014)	16	0	16	0	Centre cross fixation and sad mood inducement	Cue words – novel (positive)
Gillard et al. (2023)	21	18	0	0	30 seconds closed-eye rest	Pre-recorded voice recording of their AM memories (neutral, social inclusion and social rejection)
Keedwell et al. (2005)	12	12	0	0	Happy, sad and neutral facial expressions	Pre-recorded voice recording of their AM memories
MacDonald et al. (2016)	21	0	0	16	30 seconds rest	Cue sentences – rehearsed
Parlar et al. (2018)	12	12	0	0	Odd number detection task	Visual cues with cue words – novel
Whalley et al. (2012)	15	15	0	0	Cue words and sentences – rehearsed (identify if it is from someone else’s narrative)	Cue words and sentences – rehearsed (identify if it is from their own narrative)
Wu et al. (2020)	18	16	0	0	Visual neutral photographs displayed	Visual cues – novel
Young et al. (2012)	14	12	0	0	Subtraction task	Cue words – novel (positive, negative, and neutral)
Young et al. (2013), Young, Bodurka, et al. (2016)	16	16	0	16	Semantic example generation tasks from positive, negative, and neutral cue words and riser detection task for non-word letter strings	Cue words – novel (positive, negative, and neutral)
Young et al. (2014)	16	16	16	0	Semantic example generation tasks from positive, negative, and neutral cue words	Cue words – novel (positive, negative, and neutral)
Young et al. (2015)	20	0	20	20	Semantic example generation tasks from positive, negative, and neutral cue words and riser detection task for nonword letter strings	Cue words – novel (positive, negative, and neutral)
Young, Seigle, et al. (2016)	60	45	25	30	Riser detection task for nonword letter strings	Cue words – novel (positive, negative, and neutral)
Young, Bodurka, et al. (2017), Young, Siegle, et al. (2017)	40	40	0	0	Semantic example generation tasks from positive, negative, and neutral cue words	Cue words – novel (positive, negative, and neutral)

*Note*: HC, healthy control; HR, high-risk; MDD, major depressive disorder; rMDD, remitted major depression disorder.

### Main meta-analytic findings

**Figure 2.**
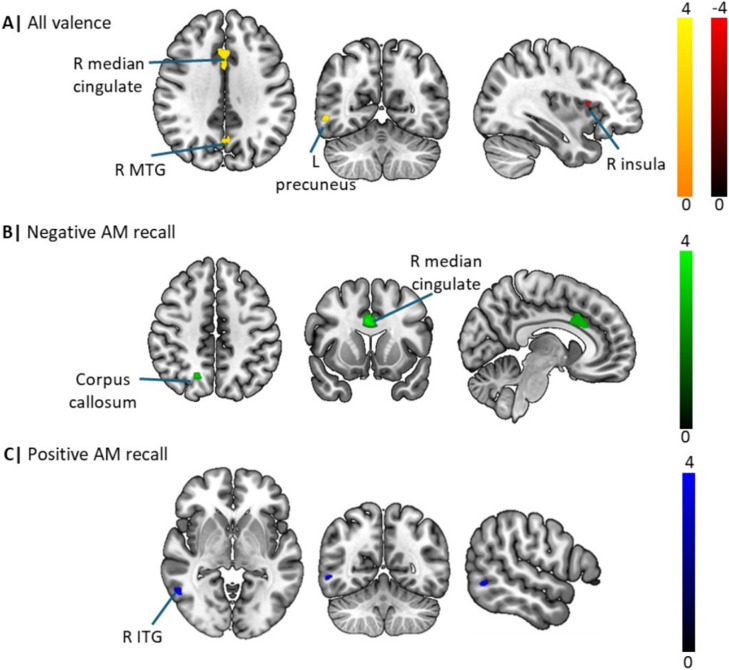
Illustration of the main meta-analytic findings. (A) Meta-analysis of all valence studies. (B) and (C) Meta-analysis of negative and positive autobiographical memory studies, respectively.

The overall meta-analysis encompassing both positive and negative valence AM recall revealed three significant clusters of increased activation in depression relative to controls located in the right anterior cingulate/paracingulate, right precuneus and the right middle temporal gyrus, and a single cluster of decreased activity in the right anterior insula. Sub-group meta-analyses investigating the effects of negative and positive valence AM recall further demonstrated that patients exhibited increased activity in the cingulate/paracingulate cingulate and right precuneus during negative recall but increased activity in the right inferior temporal gyrus during positive recall (Figure 2; Table 2).

### Functional connectivity and behavioral-level analyses

**Figure 3.**
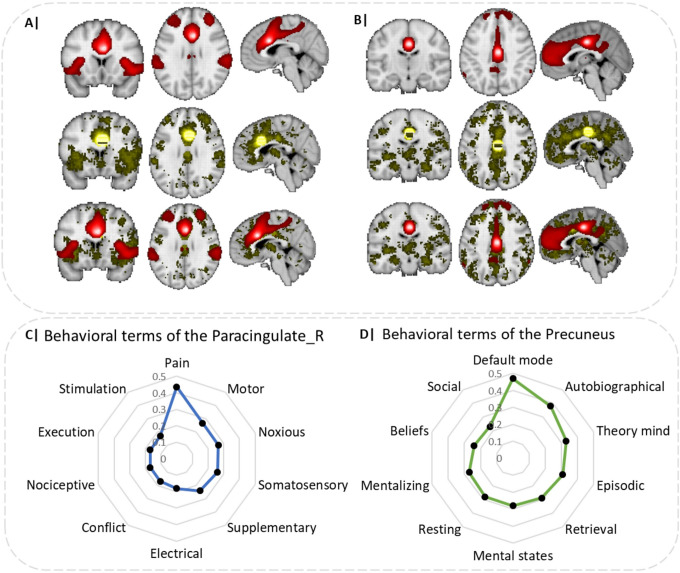
(A) and (B) Functional connectivity and meta-analytic co-activation of the regions identified of being altered in depression during AM recall for paracingulate regions and precuneus. The top panel shows the functional connectivity, the middle panel shows the meta-analytic co-activation patterns, and the bottom panel shows the combination of both functional and meta-analytic co-activation patterns. Behavioral terms of the paracingulate and precuneus are shown in (C) and (D), respectively.

Additional analyses were conducted on the network and behavioral levels. Network analyses were performed utilizing the Neurosynth database (https://www.neurosynth.org/) to explore the functional connectivity and meta-analytic coactivation patterns of the paracingulate and precuneus regions identified in the general valence meta-analysis as shown in Figure 3A and B, respectively. The top panel shows the strong functional connectivity of the paracingulate with core regions of the salience network, including the ACC as well as the bilateral anterior insula (extending into the inferior frontal gyrus). The overlap of the functional connectivity and meta-analytic coactivation maps confirmed that core connectivity profiles encompassed the anterior insula and anterior cingulate cortex (ACC; Figure 3A). The functional connectivity for the seed voxel in the precuneus showed interactions with core cortical midline regions of the default mode network, in particular medial frontal regions as well as the precuneus and PCC. The overlap of the functional connectivity and meta-analytic coactivation maps confirmed functional interactions with the cortical midline regions (Figure 3B).

The additional behavioral characterization indicated that the paracingulate region is strongly associated with negative affective (eg, pain) and executive (conflict) functions, while the precuneus regions is associated with social cognitive and mnemonic functions, including autobiographic and theory (of) mind as shown in Figure 3C and D, respectively.

### Additional analyses: Meta-regression

Meta-regression analyses of age and gender as possible confounding variables were conducted. The analysis found no significant associations.

### Tests for heterogeneity and publication bias

Between-study heterogeneity was generally low across the three analyses. With reference to the bias p-values and symmetric funnel plots, publication bias analysis was not statistically significant as shown by smaller p-values in the Egger’s p column, of which the test significance threshold was set to p < .05 (see Table 2).

**Table 2.** Meta-analytic results of depression patients versus healthy controls in autobiographical memory recall at p < .0025 uncorrected

Meta-analysis	Anatomical region (s)	Voxels	MNI coordinates	Z value	Hedges’ g	I^2^ (%)	Eggers’ bias	Eggers’ p
Valence AM recall	R median cingulate/paracingulate gyri, BA 24	344	4, 16, 28	4.021	0.4	4.09	0.19	0.88
	R precuneus	40	2, −68, 38	3.221	0.37	10.06	0.97	0.46
	R middle temporal gyrus	37	56, −56, −2	3.456	0.37	3.62	0.81	0.56
	R insula	11	36,12,8	−3.307	−0.35	8.23	−0.82	0.59
Positive valence	R inferior temporal gyrus	26	56, −58, −4	3.207	0.4	11.43	0.83	0.62
Negative valence	Right median cingulate/ paracingulate gyri	168	4, 14, 30	3.712	0.45	3.69	0.95	0.56
	Corpus callosum	11	18, −62, 44	3.101	0.37	0.92	0.1	0.95

*Note*: AM, autobiographical memory; L, left; MNI, Montreal Neurological Institute; R, right.

## Discussion

Our main meta-analysis identified increased activation in the right median cingulate/paracingulate gyri (overlapping with the dACC), left precuneus and right inferior temporal gyrus and concomitantly increased activity in the right anterior insula in depressive individuals compared with healthy controls during AM recall. These results were consistent when running a separate analysis excluding the at-risk individuals suggesting that these do not bias the estimation. Moreover, exploratory valence-specific meta-analyses revealed increased activity in an overlapping network during negative recall (ie, dACC and precuneus) and positive recall (inferior temporal gyrus) suggesting potentially valence-specific effects. On the network level, the core regions encompassing the dACC and precuneus exhibited functional interactions with the salience network and the DMN, respectively. Behavioral characterization further revealed an engagement in negative affect and executive functions of the dACC and AM and social cognition of the identifies precuneus region respectively.

While the functional and behavioral characterization aligns with the corresponding deficits in these domains in depression (eg, Xu et al., 2022; Young et al., 2012), the identified regions did not align with our hypothesized alterations, including reduced activation of the hippocampus, and increased activation of the amygdala during AM in depression. Both of these neural alterations have been conceptualized and integrated into memory and cognitive theories on depression. For instance, overarching theories posit that the valence-specific memory deficits in depression are related to stress-associated suppression of hippocampal functioning and sensitization of the amygdala (Dillon & Pizzagalli, 2018). Moreover, the cognitive reactivity posits that sadness or stress activates dysfunctional cognitive schemata in depressed patients, which in turn maintains depressive processing styles (Lau et al., 2004) via the DMN and the hippocampus (Marchetti et al., 2012; Mulders et al., 2015). Despite the conceptual framework results in original fMRI studies on AM were not unequivocal and may depend on the analyses (region focused versus whole-brain), valence of the tasks (ie, recalling positive, negative, and neutral AM), as well as ambiguity in certain paradigms (ie, recalling specific or any AM). While some of the original studies reported elevated amygdala activity and greater amygdala-hippocampal connectivity when recalling negative AM (Doré et al., 2018; Young et al., 2014; Young, Bodurka, et al., 2016; Young, Siegle, et al., 2016), these effects may not have been robust enough to generate consistent results across the heterogenous studies. Furthermore, an increasing number of recent studies challenges the valence-specific function of the amygdala, indicating rather a general role in arousal or salience (Zhang et al., 2025) and recent large-scale meta-analyses reported null findings in tasks traditionally related to amygdala activation, such as the emotional Stroop task (Chen et al., 2018; Feng et al., 2018).

The identified dACC region in the present study has traditionally not been considered to play a key role in AM per se. In the context of mnemonic functions this region has been associated with remote AM recall, which together with a recent meta-analysis suggests a potential contribution to AM (Daviddi et al., 2023; Steinvorth et al., 2006) (see also Martinelli et al., 2013). However, in line with the meta-analytic functional and network-level characterization the dACC plays a key role in affective and executive functions, including conflict processing and negative emotional appraisal (Etkin et al., 2011; Li et al., 2020), with recent work from Gillard et al. (2023), further suggesting that the dACC could be linked to activations across the affective salience network as a response to the psychological experience of social pain and affiliation (Dalgleish et al., 2017; Eisenberger et al., 2003), which aligns with previous findings indicating altered activity in this region during negative AM recall in depression (Young, Bodurka, & Drevets, 2017).

Previous studies on dACC in depression have reported robust alterations in the functional intrinsic network architecture of this region (Zhou et al., 2024) and an association with different symptom domains in depression (Sheline et al., 2010). During task engagement this region has demonstrated altered functional communication during social cognitive processes, including, for example, social working memory (Xu et al., 2022), which together with the stronger contribution of this region in the negative AM studies on depression may reflect that dysregulations in this area may contribute to an overgeneralized memory in depression (Falco et al., 2015; Raes et al., 2023) or mediate affective emotional aspects as well as general cognitive deficits in depression leading to emotionally biased re-experience of social affective experiences (Gillard et al., 2023).

The precuneus has been strongly associated with AM retrieval, with research on the specific functions indicating a contribution to autobiographical reminiscence (Daviddi et al., 2023; Shepardson et al., 2023; Sreekumar et al., 2018), or first person visual and memory-based navigation (Wilson et al., 2005). It further shows its association with self-referential processing and self-consciousness (Freton et al., 2014). Interestingly, Freton et al. (2014) found that the spontaneous tendency to recall AM is positively correlated to precuneus volume which may imply a contribution to spontaneous AM retrieval. Within recent pathological models of negative emotional experiences, for example, in Post Traumatic Stress Disorder, posteromedial regions such as the precuneus have been related to dysregulations in visual imagery processes (Thome et al., 2020). The contribution of this region to autobiographical, self-referential processing, and social cognitive functions is further underscored by or behavioral characterization and the meta-analytic characterization as central part of the default mode network (see also Xin et al., 2021). Together the findings may reflect that alterations in this region may mediate the mnemonic deficits in the patients, potentially reflecting overgeneralized memory recall or dysfunctional imaginary and self-referential processes in depression. Decreased spontaneous activity in the precuneus has been demonstrated in depression – yet not related disorders such as bipolar disorder (Gong et al., 2020) – with recent evidence indicating a role of altered precuneus engagement during aberrant self-referential AM processing adolescents with depression (van Houtum et al., 2023) as well as less vividness in AM retrieval in depression (van Schie et al., 2019). This further supports the notion that depressed individuals are less likely to retrieve vivid AM and may do so in the context of aberrant self-referential appraisal.

The third significant cluster of increased activation was in the inferior temporal gyrus, a subregion of the DMN. Although the specific role of the inferior temporal gyrus is not well explored, Sheline et al. (2009) proposed that depressed individuals do not inhibit DMN activity while looking at negative pictures, which could possibly be linked to negatively valenced material. Lemogne et al. (2012) further supported this finding by indicating that depressive self-focus was related to the lack of DMN inhibition. This implies that there are underlying mechanisms regarding depressive individuals’ possible fixation on negative AM contributing to the maintainence of depressive symptoms.

Conversely, the SDM-PSI analysis found reduced activation of the right anterior insula. The insula is important in supporting subjective feeling states or interoceptive processes (Critchley et al., 2004; Zhang et al., 2023) and more vivid memories are associated with the activation of the insula (van Schie et al., 2019). With this in mind, it could be proposed that depressive patients are more likely to have reduced vividness in AM recall, which is indicative of decreased awareness of oneself. This implies that depressive patients are more distant when recalling AM, which is supported by Beck (1979), who posits that distancing oneself from a dissonant past self, or evaluating the self with a distance, are processes that are central to proposed theoretical accounts of depression. This was also supported by a study led by Kuyken and Howell (2006), which found that depressive patients have a more distanced view of their AM as compared to healthy individuals. The decreased activation of the insula could further suggest that depressive patients are more likely to have lowered awareness of the self, and an affected AM recall.

Functional connectivity analysis of the seed voxel in the paracingulate identified associations with the brain regions in the salience network, including the anterior insula, and the ACC (Uddin et al., 2019). The anterior insula plays an important role in supporting both, subjective feeling states of emotional experience and autonomic reactivity (Ferraro et al., 2022) which may reflect that depressive individuals are more likely to fixate on negative emotional and autonomic experiences of AM (Dillon & Pizzagalli, 2018). In line with this interpretation previous studies indicate an engagement of the insula during recollection or reflection on personal distress (Eisenberger, Lieberman, & Williams, 2003; Wager et al., 2003) and evaluation of negative emotional states (Sanfey et al., 2003). Associations with the ACC supports previous research proposing that AM retrieval relates to subjectively painful experiences (Dalgleish et al., 2017; Eisenberger et al., 2003; Kelly et al., 2007). Together, the involvement of the salience network may reflect a more aversive affective experience during AM in depression or a dysregulated attentional balance between internally and externally focused attention (Gillard et al., 2023; Xin et al., 2021).

Furthermore, the functional connectivity analysis for the seed voxel in the precuneus points to correlations to the DMN. The DMN, more specifically the anterior medial PFC (amPFC) and the PCC, is crucial in AM retrieval as it facilitates self-referential processing (Andrews-Hanna et al., 2014). Individuals with MDD, or individuals with high risk of developing depression show alterations in the activation of DMN (Chou et al., 2023; Posner et al., 2016). Depressed individuals tend to show differential engagement in the DMN and self-referential processes during task and rest conditions (Chou et al., 2023). Due to its core function and possibly causal contribution to free but not cued AM (Bonnici et al., 2018; Lanius et al., 2020), alterations in the DMN may reflect impaired-free AM recall in depression. Finally, the corresponding behavioral characterization supports the engagement of both large-scale networks with the large-scale networks and suggests that dysbalanced engagement of the salience network and DMN facilitate AM deficits in depression.

Findings need to be considered in the context of limitations. First, several studies utilize specific AM recall (Young et al., 2012, 2013, 2014, 2015; Young, Bodurka, et al., 2016; Young, Siegle, et al., 2016), posing difficulties in determining the valence of AM retrieved. While we aimed at disentangling valence-specific effects utilization of the valence-specific results should be interpreted with caution due to the comparably low number of studies (Dahlgren et al., 2020; Ding et al., 2022). Second, there has been increasing support of semantic and episodic AM retrieval having independent neural networks and brain regions involved (Levine et al., 2004; Maguire et al., 2000), and an increasing number of studies meta-analyses can focus on depression-related dysfunctions in episodic and semantic AM components. The studies were mostly conducted in western populations (exception: Wu et al. (2020) with participants from Southwest China). Studies have proposed that cultural contexts modulate the development of episodic AM (Harbus, 2010), and an increasing number of studies employ a cross-cultural neuroimaging approach (Yang et al., 2023).

In summary, the present meta-analysis does not support prevailing models that suggest amygdala and hippocampal engagement underlying AM deficits in depression. Rather, results support the role of core nodes in the salience (dACC) and default mode network (precuneus) as a pathological substrate of AM dysfunctions among people with depression or those at risk. With the dACC being linked to affective and executive functions, results suggest that its activation could be associated with activations in the salience network as a response to the psychological experience of social pain and affiliation. Findings indicate that activation in the precuneus is in line with current research, further emphasizing its role in AM retrieval, specifically in overgeneralised memory recall or dysfunctional imaginary and self-referential processes in depression.

## Supporting information

10.1017/S0033291725102833.sm001 Cheng et al. supplementary material Cheng et al. supplementary material

## Data Availability

All included studies have been cited within the paper. Data supporting the results of this paper are available at the Open Science Repository platform (OSF) (https://osf.io/35xtf/).

## Supplementary material

The supplementary material for this article can be found at http://doi.org/10.1017/S0033291725102833.
